# *Yersinia pestis* Genotyping

**DOI:** 10.3201/eid1108.040942

**Published:** 2005-08

**Authors:** Gilles Vergnaud

**Affiliations:** * Centre d'Etudes du Bouchet, Vert le Petit, France

**Keywords:** Ancient DNA, plague, Yersinia pestis, tandem repeats, MLVA, pandemics

**To the Editor:** Drancourt et al. (1) report the development of an original genotyping system for *Yersinia pestis* based on intergenic spacer sequencing. However, the approach appears to rely upon the characterization of polymorphisms due to tandem repeat variation. Eight spacers are used in the report, 7 of which contain tandem repeats, and the sequence variability used to produce the typing data and the strain clustering result from variation in the number of tandem repeats (and incorrect data analysis produces a dendrogram with 34 branches from only 19 different isolate types). Three of the spacers and associated polymorphisms were previously reported. Spacers YP3 and YP5 are, respectively, ms38 and ms56 (2); spacer YP10 is M61 (3). YP3 is later used to investigate ancient DNA samples, and 3 amplification products are described in detail. The sequences are compared to modern sequences by BLAST analysis, which is not relevant for tandem repeats. Instead, the Figure shows how internal variation within the array can be coded to facilitate interpretation. In this collection, Orientalis strains are "abcdeeef," whereas Antiqua strains from Africa are "abcdeef." All these different codes can be deduced one from the other by simple duplication and deletion events, with no need to invoke point mutations. The codes for all 3 ancient samples are identical to the Orientalis code "abcdeeef."

**Figure:**
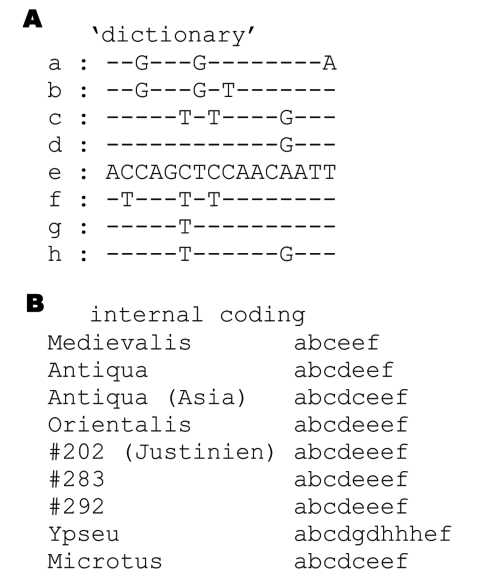
A) sequence-to-code correspondence (1 letter per 16-bp repeat unit). Differences from repeat unit "e" are shown. B) Tandem repeat arrays were coded accordingly. All sequences were obtained from Genbank (Ypseu: Yersinia pseudotuberculosis IP32953; Microtus: "Y. microtus" Chinese strain #91001).

In conclusion, the data presented by Drancourt et al. do not appear to support their claim. They did not invent a new genotyping method but used the well-known multiple locus variable analysis (MLVA) number of tandem repeats approach. The finding that the "genotype Orientalis was involved in all three pandemics" is not valid since the Orientalis type is defined by a biochemical assay, resulting in all known Orientalis strains from a 93-bp glycerol-3-phosphate dehydrogenase microdeletion (4,5), which was not investigated here.
