# Supplementary material for: Yersinia pestis Genotyping
Source: Emerg Infect Dis. 2005 Aug;11(8):1317–9. doi: 10.3201/eid1108.040942 (PMC3320496; doi:10.3201/eid1108.040942)
Supplement: Related texts

# Reply: *Yersinia pestis* Genotyping

**DOI:** 10.3201/eid1108.050568

**Authors:** Didier Raoult, Michel Drancourt, Pierre Edouard Fournier, Hiro Ogata

**Affiliations:** * Centre National de Reference, Marseille, France

**In Response:** We thank Dr. Vergnaud for his response (1). Since the time of our publication (2), 2 articles related to our paper were either submitted or published. One (3) reported identification of *Yersinia pestis*–specific genes in teeth from patients who died during the Justinian plague; another proposed identification of *Y. pestis* strains by using variable numbers of tandem repeats analysis (VNTR) (4). The authors concluded that isolates could easily be compared in their database by using 7 markers. As opposed to work with cultures where ample, high-quality DNA template is available, successful amplifications with 7 different primer sets cannot be achieved by using DNA extracted from ancient teeth (5). By comparing genome sequences, we evaluated short intergenic spacers that were more divergent. Divergences included mutations, deletions, and duplications (VNTR). Phylogenetically, an entire repeat unit has the same weight as that of a single nucleotide polymorphism. By sequencing, we have identified all events (single nucleotide polymorphism and VNTR). Sequencing is more versatile for use in strain identification (5), allows distinction at the species level, and can be applied directly on clinical and forensic samples.

The discovery of a unique sequence is critical to authenticate results in such controversial areas as paleomicrobiology (5). Fortunately, we have identified a unique sequence that contains several mutations. These mutations do not exclude this strain from being *Y. pestis* (see Figure). Additionally, we doubt that our conclusions would have been accepted had we simply used the VNTR, demonstrating only an amplicon of the right size on a gel.

**Figure:** Unrooted tree showing the phylogenetic relationships between the sequence obtained from the YP3 spacer from Justinian sample 202 and that from the genomes of *Yersinia pestis* strain CO92 (GenBank accession no. AJ414159), *Y. pestis* biovar Medievalis (AE017139), *Y. pestis* strain Kim (AE013993), and *Y. pseudotuberculosis* (BX936398). DNA sequences were aligned by using the ClustalW software, version 1.81 (2). Deletions were considered single events. A distance matrix was constructed by using the Kimura-2 parameter, and the phylogenetic tree was inferred by using the neighbor-joining method in the Mega2 software package. The scale bar represents a 0.5% nucleotide sequence divergence. Bootstrap values are indicated at the nodes (2).

In conclusion, our results have been validated by others. The sequence is original and, therefore, authentic. Dr. Vergnaud agrees that the results we presented did represent a sequence associated with the Orientalis biovar. This finding may end the controversy.

## References

[1] Vergnaud G. *Yersinia pestis* genotyping. Emerg Infect Dis. 2005;11:1317–8.1611058310.3201/eid1108.040942PMC3320496

[2] Drancourt M, Roux V, Dang LV, Tran-Hung L, Castex D, Chenal-Francisque V, Genotyping, Orientalis-like *Yersinia pestis*, and plague pandemics. Emerg Infect Dis. 2004;10:1585–92.1549816010.3201/eid1009.030933PMC3320270

[3] Weichmann I, Grupe G. Detection of *Yersinia pestis* DNA in two early medieval skeletal finds from Aschheim (Upper Bavaria, 6th century AD). Am J Phys Anthropol. 2005;126:48–55. 10.1002/ajpa.1027615386257

[4] Pourcel C, Andre-Mazeaud F, Neubauer H, Ramisse F, Vergnaud G. Tandem repeats analysis for the high resolution phylogenetic analysis of *Yersinia pestis*. BMC Microbiol. 2004;4:22. 10.1186/1471-2180-4-2215186506PMC436057

[5] Drancourt M, Raoult D. Paleomicrobiology: current issues and perspectives. Nat Rev Microbiol. 2005;3:23–35. 10.1038/nrmicro106315608697
